# Adding tactile feedback increases avatar ownership and makes virtual reality more effective at reducing pain in a randomized crossover study

**DOI:** 10.1038/s41598-023-31038-4

**Published:** 2023-05-22

**Authors:** Hunter G. Hoffman, Miles R. Fontenot, Azucena Garcia-Palacios, Walter J. Greenleaf, Wadee Alhalabi, Michele Curatolo, Herta Flor

**Affiliations:** 1 Virtual Reality Research Center, Mechanical Engineering, University of Washington, Seattle, 98195 USA; 2 Department of Anesthesiology and Pain Medicine, University of Washington, Seattle, 98195 USA; 3 Department of Basic Psychology, Clinic and Psychobiology, Jaume I University, 12071 Castellón de La Plana, Spain; 4 Virtual Human Interaction Lab, Stanford University, Stanford, 94305 USA; 5 Department of Computer Science, Faculty of Computing and Information Technology, King Abdulaziz University, Jeddah, 21589 Saudi Arabia; 6 Department of Cognitive and Clinical Neuroscience, Central Institute of Mental Health, Medical Faculty Mannheim, Heidelberg University, 68159 Mannheim, Germany

**Keywords:** Psychology, Health care

## Abstract

Severe pain is a widespread health problem in need of novel treatment approaches. In the current study we used real water to give virtual objects (i.e., animated virtual water) more realistic physical properties (wet liquid qualities). Healthy volunteers aged 18–34 participated in a within-subject randomized study comparing participants’ worst pain during brief thermal stimuli with (1) No Immersive Virtual Reality (VR), versus (2) during VR + no tactile feedback versus (3) VR + real water (with tactile feedback from co-located real objects). Tactile feedback significantly decreased pain intensity (VR analgesia, *p* < 0.01), compared to VR with no tactile feedback, and compared to No VR (baseline). Tactile feedback made the virtual water feel significantly more real, increased participant’s sense of presence, and both VR conditions were distracting (significantly reduced accuracy on an attention demanding task). As a non-pharmacologic analgesic, mixed reality reduced pain by 35% in the current study, comparable to the analgesia from a moderate dose of hydromorphone in previous published experimental studies. Tactile feedback also significantly increased avatar embodiment, the participants illusion of ownership of the virtual hands, which has potential to improve the effectiveness of avatar therapy for chronic pain in future studies. Mixed reality should be tested as treatment in pain patients.

## Introduction

Excessive pain during medical procedures is a worldwide medical and healthcare/psychological problem, for example, pediatric burn wound care^1^. Opioid side effects limit dose levels and limit analgesic effectiveness, and in the USA, new more restrictive federal regulations now reduce prescriptions for opioids, in light of the current opioid overdose epidemic^2^. Repeatedly enduring severely painful wound care sessions (e.g., burn wound debridement) increases the patients’ risk of developing chronic pain. In some cases, the patients’ experiences during painful burn wound care sessions turn into traumatic memories. Children may have nightmares about their burns and wound care, and other Post-Traumatic Stress Disorder symptoms that can persist for years after the physical wounds have healed^3^.

A number of psychological factors such as catastrophizing^4^, expectations of pain, anxiety, and memory for previous painful events, can amplify how much pain patients experience. For example, a patient having a mild panic attack as they are being brought into the hydrotank room for burn wound care, will likely require strong analgesics and sedation, and will be more challenging to keep comfortable during a painful medical procedure^5–9^. Psychological treatments such as meditation, cognitive behavioral therapy, and distraction^10^ can help reduce the experience of pain. Immersive Virtual Reality (hereafter referred to as VR) analgesia, first introduced in the late 1990s^11,12^ is a very effective computer-generated distraction technique powerful enough to help reduce severe pain during burn wound debridement^1,13^. VR has now been shown to help reduce acute pain during a wide range of painful medical procedures such as dental procedures, venipuncture, physical therapy, endoscopic surgery, and orthopedic pin removals from healed pelvic bones^14^ see^15,16^ for reviews.

During burn wound care with immersive VR, a Head Mounted Display, (HMD) blocks the patient’s view of the real world, so the patient is not able to see the hospital room and is not able to watch their wound care. Instead, patients see and interact with a pleasant computer-generated environment (e.g., SnowWorld) that is completely unrelated to their injury and unrelated to their painful medical procedure, allowing patients to mentally “escape” from their pain into the pleasant computer generated world. We theorize that during VR, multisensory computer generated information floods into the patients’ brain through their sensory systems (e.g., the visual cortex) and interferes with the brain’s conversion of nociceptive signals into the patients’ conscious experience of pain, reducing pain-related brain activity, and reducing the perception of pain. Children recovering from large severe burn injuries report large reductions in pain while they are in VR, the patients indicate spending less time thinking about their pain during VR, and even report having fun during wound care while in virtual reality^1^. Functional magnetic resonance imaging (fMRI) has been used to assess the brain correlates of VR-induced pain reduction, and research has shown that VR analgesia involves significant reductions in activation in brain regions such as the anterior cingulate cortex, insula, thalamus and the primary and the secondary somatosensory cortices^17^. Another fMRI study comparing VR analgesia to opioid analgesia showed that VR reduces pain as much as a moderate dose of hydromorphone^18^ with similar changes in brain activation.

The essence of immersive virtual reality is the participants’ illusion of “being there” in the computer generated environment, as if the virtual world is a place they are visiting, an illusion known as “sense of presence”. It is assumed that a stronger illusion of presence is associated with more attentional resources being drawn into the virtual world, leaving less attention available to process nociceptive signals, thus reducing the perceived experience of pain (i.e., an attentional mechanism). Although the mechanism of how VR reduces pain is not well understood, consistent with the hypothesis of an attentional mechanism, experimental studies have shown that an increase in the immersiveness of the VR system (e.g. increasing the field of view participants see through the HMD, increasing interactivity and/or adding avatars) can significantly amplify how much VR reduces pain^19,20^, and increasing interactivity often also enhances how “present” people feel in the virtual world^21,22^.

The current experimental pain study with healthy volunteers explored whether adding physical properties (wet physical liquid tactile sensations) to virtual objects (computer generated animated water seen through the HMD) would increase the analgesic effectiveness of virtual reality as a non-opioid pain reduction technique. Immersive VR mixed reality techniques involving co-locating real and virtual objects (i.e. tactile augmentation)^11,23^ have previously been shown to make virtual objects more compelling and more real, via converging multisensory input from sight and touch (e.g., using a position tracked ceramic plate to make a virtual plate feel more solid, heavy and real)^11,23^. We predicted that similarly, in the current study, the participants’ visually dominant brain would unify the sensory input from the visual and tactile senses, and participants would have the compelling illusion of physically touching the computer generated virtual water they saw visually through the HMD.

In the current study, using a camera-based hand tracking system with a first person perspective, participant’s real hand movements were mimicked by their corresponding “visual only” virtual avatar hands seen through the VR Head Mounted Display (HMD). In addition to increasing VR analgesia, we assumed that participants would report a stronger illusion of presence, and greater ownership/embodiment of their virtual avatar hands during the more immersive “mixed reality” (avatar VR + real water), compared to touching virtual water only (no real water/no tactile feedback) and we predicted a significant correlation between sense of presence in VR, and the amount of pain reduction during VR with tactile feedback. With respect to other mediating factors, we predicted that participants would make more errors on a divided attention task during VR, (an objective measure of how much attention VR diverts) and we predicted that these errors would be enhanced in the mixed reality VR condition. Moreover, we predicted that conditioned pain modulation, an important measure of endogenous pain inhibition, would be related to analgesia when participants were immersed in the virtual reality scenario and were receiving the tactile feedback from the virtual water (i.e., analgesia during the VR + real water condition).

## Method

**Figure 1:**
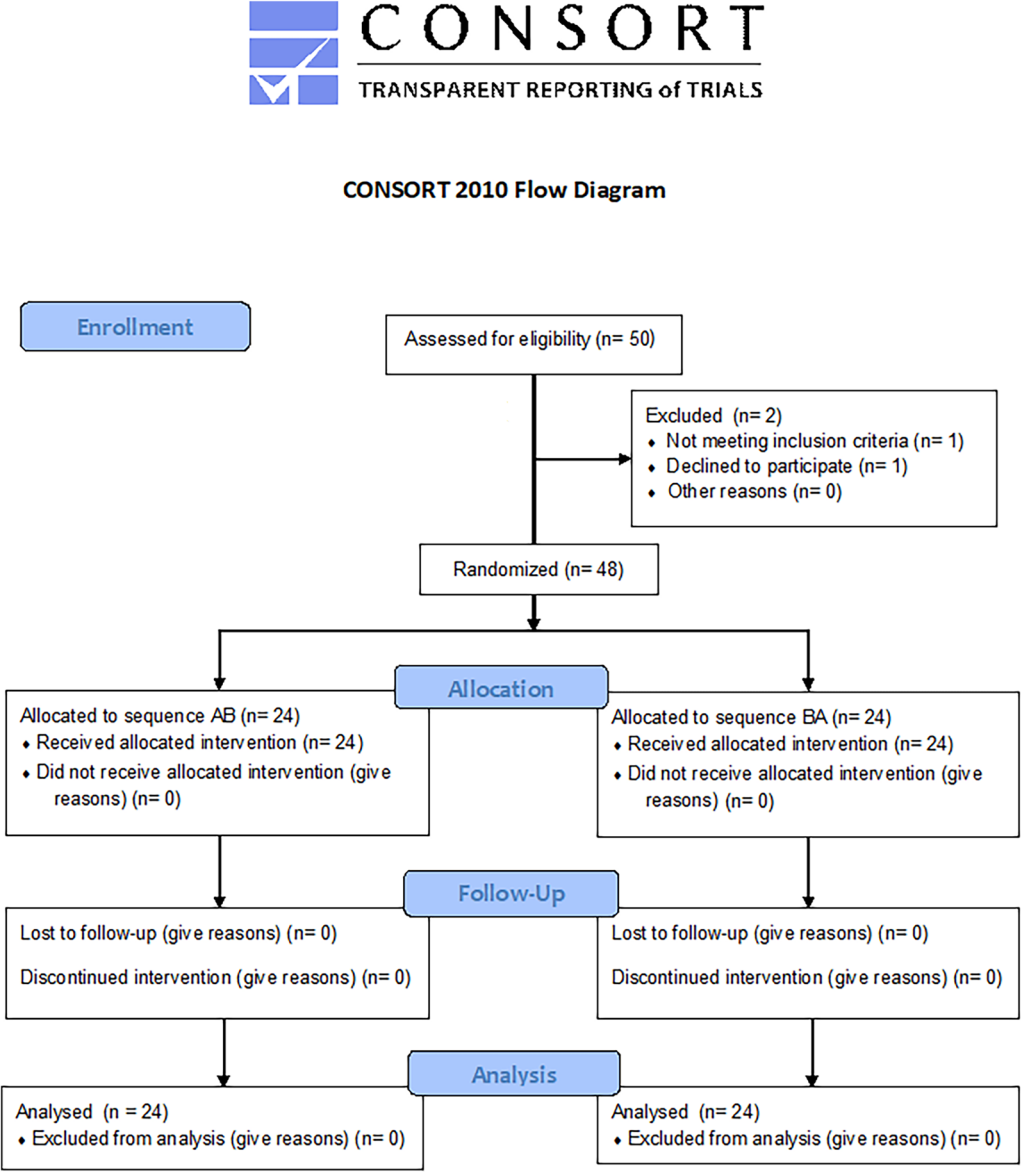
Consort 2010 Flowchart.

Forty-eight healthy undergraduate psychology students from the University of Washington, Seattle participated in the study between April 25, 2022 and June 3, 2022. The characteristics of the participants are shown in Table 1. Ethical committee approval was obtained from the Institutional Review Board of the University of Washington Committee B, and written informed consent was received from all participants. The study protocol adhered to the Declaration of Helsinki and the study was registered at ClinicalTrials.gov (Identifier: NCT05130307, date of registration: 23/11/2021 ). The inclusion and exclusion criterion are shown below. See participant enrollment Consort 2010 Flowchart in Fig. 1. The data was collected in an experiment room in Guthrie Hall, Psychology Department, University of Washington, Seattle.

**Table 1:** Characteristics of the participants.

Ethnicity	Asian = 69%	African American = 2%	Caucasian = 29%
Age range 18–34 years old, Mean age = 19.60, SD = 2.48			
Gender, 54% M, 46% female

Inclusion Criteria:

- Currently enrolled in a course at the University of Washington Psychology Dept., participating in the UW Psychology subject pool
- Able to read, write and comprehend English
- Able to complete study measures
- Willing to follow our UW approved instructions
- 18 years of age or older

Exclusion Criteria:

- People how have already previously participated in this same study (e.g., last quarter) are not eligible to participate again.
- Not enrolled in a course at the University of Washington Psychology Dept., participating in the UW Psychology subject pool
- Not be able to read, write and comprehend English
- Younger than 18 years of age.
- Not capable of completing measures
- Not capable of indicating pain intensity,
- Not capable of filling out study measures,
- Extreme susceptibility to motion sickness,
- Seizure history,
- Unusual sensitivity or lack of sensitivity to pain,
- Sensitive skin,
- Sensitive feet
- Migraines
- Diabetes

### Equipment

The VR system was an untethered self-contained battery powered Quest2 stand alone wireless HMD VR system, with optical hand tracking (Meta, Menlo Park, California, https://www.Meta.com). The VR software used was a commercially available VR game named Waltz of the Wizards (Aldin Dynamics, Reykjavik, Iceland, https://www.aldin.io/). In both VR treatment conditions, each subject could see a medieval room in a castle in virtual reality, with a large virtual wooden bowl full of computer-generated animated virtual water within reach of their virtual hand. No audio or sound effects were used in the current study (audio volume of the HMD was set at zero). Participants were informed that they would interact with virtual objects briefly during different phases of the study, but were not told what results were expected.

### Procedures and design

#### Within-subjects design

**Figure 2:**
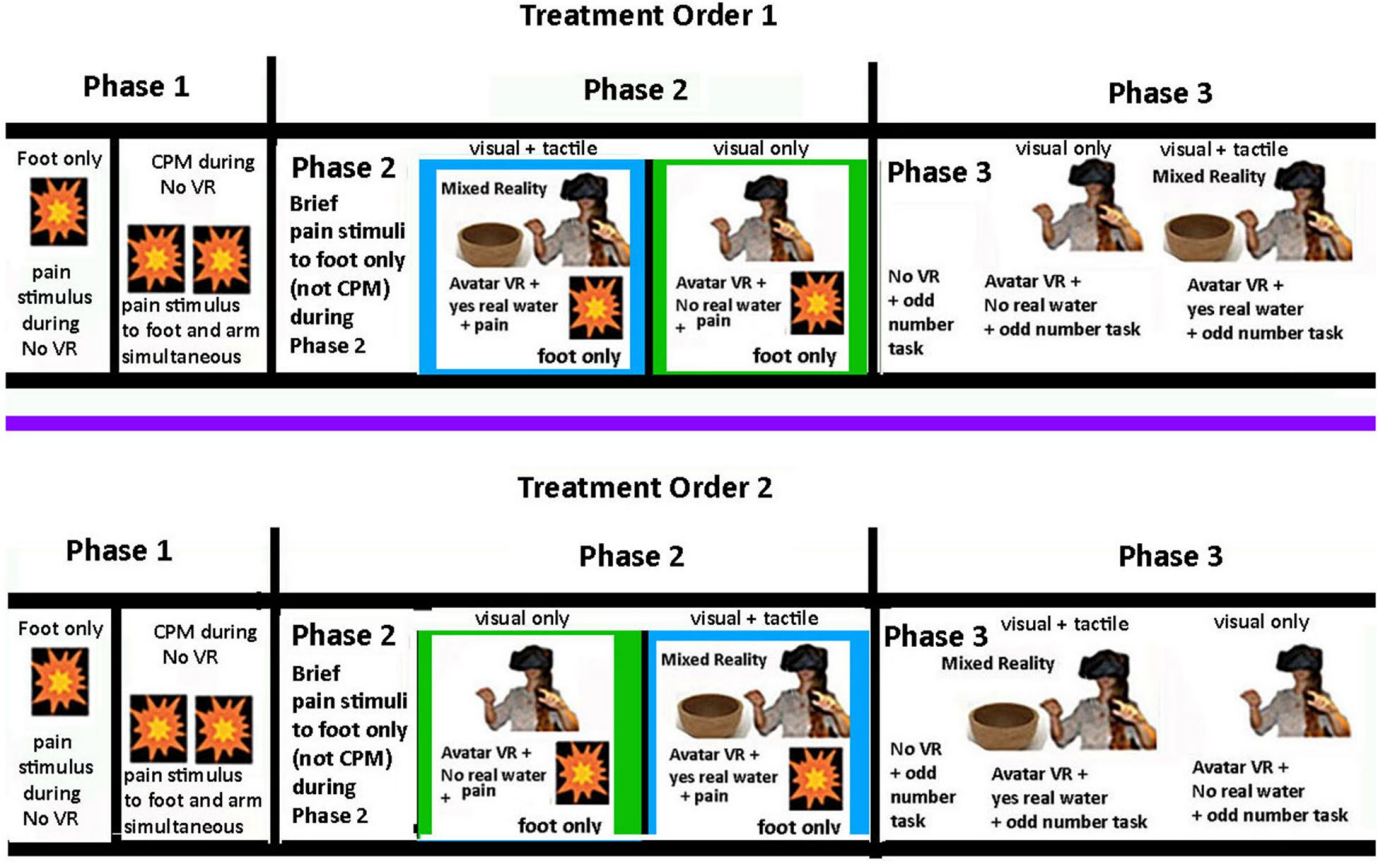
A detailed description of the stimulus sequence. Participants were randomly assigned to either Treatment Order 1 or Treatment Order 2, with VR treatment order randomized).

During the ”VR with No real water” condition, as participants put their virtual hand into the bowl of computer generated virtual water in virtual reality, their real hand reached into the air in the real world (no tactile feedback, i.e., no real water). In contrast, during the “VR with Yes real water” condition with tactile feedback, when the participant put their virtual hand into the bowl of virtual water in VR, their real hand simultaneously reached into a co-located bowl of real water in the real world (VR + real water). Using an AB/BA within subject crossover design with treatment order randomized, half of the participants received “VR + yes real water” first and VR + no real water” second, and others received the opposite order “VR + No real water ” first and “VR + Yes real water” second. This within-subjects design permitted a comparison of both conditions within the same subject, which made the design statistically more powerful^24^ by reducing nuisance variance. The protocol used in the current study is depicted in Fig. 2.

### Phase 1: quantitative sensory testing

Adapting the paradigm used by our team in several previous studies^17,18,22^, in the current study, a commercially available Medoc Quantitative Sensory Testing Q-Sense CPM thermal (heat) pain stimulator (Medoc, Ramat Yishay, Israel) (Ramp and Hold program) was used to deliver very brief heat stimuli to the top of the participant’s left foot, at a “painful but tolerable” temperature individually pre-selected and pre-approved by each participant. The overall duration of the heat lasted several seconds for each stimulus, increasing at 1 °C per second. The thermode temperature began to return to baseline temperature immediately after reaching the programmed peak at a rate of 1 °C per second. The mean temperature participants selected (baseline) was 44.32 °C (SD = 1.91, range = 42–48.0 °C), and on a scale from zero to ten, the mean worst pain rating at baseline was 4.71, (SD = 1.25, range 2–8).

During Phase 1, each participant was allowed to choose the temperature they wanted to use during the test phase. During Phase 1, starting at a relatively low temperature, the participants slowly progressed to a temperature they found “painful but tolerable” for the very brief duration that they agreed to receive several more times later in the study.

#### Ratings

In this study, after each thermal pain stimulus, five separate ratings were administered (including 3 pain ratings) with a pictorial example of the labeled graphic rating scale for each query (see full questionnaire in "Appendix 1"). The scales had the endpoints “no pain” and “excruciating pain” for pain intensity, ”not unpleasant at all” and “excruciatingly unpleasant” for pain unpleasantness and ”none of the time” to “all of the time” for time spent thinking about pain. The scales were designed to capture sensory, affective and cognitive aspects of the pain experience^22,25,26^.

After each baseline pain stimulus (foot only) subjects received the following instructions prior to answering the five separate queries. “Please indicate how you felt during the most recent pain stimulus by making a mark anywhere on the line. Your response does not have to be a whole number.” After each brief thermal stimulus, participants indicated how painful they found the stimulus to their foot, using Graphic Rating Scales (GRS) to measure “worst pain”, “pain unpleasantness”, and “time spent thinking about pain”. Participants were also asked how much fun they had during the most recent thermal stimulus, and how anxious/nervous they were during the baseline pain stimuli and after each subsequent pain stimulus.

### Conditioned pain modulation

VR was not used in Phase 1. During Phase 1, after the baseline “painful but tolerable” temperature (foot only) to the dorsal (top) of the left foot was identified, a second thermode (same temperature and stimulus duration as the baseline left foot) was attached to participants’ left wrist to measure conditioned pain modulation, as an indicator of endogenous inhibitory control^27^. After an approximately 5 min interstimulus interval “washout period” (after the single thermode baseline measure of foot pain), participants then received a very brief simultaneous stimulation of their foot and wrist at the same time. Both the foot and wrist were stimulated at the “painful but tolerable” baseline temperature the participant had chosen and agreed to use. After the simultaneous stimulation to their hand and wrist, participants were again asked to rate how much pain they felt on their “foot only” during the “foot + wrist” stimuli, ignoring the pain they felt on their wrist (the conditioning stimulus). (They also rated how much pain they felt on their “wrist only” during the same “foot + wrist” stimuli, ignoring the pain they felt on their foot, but the “wrist only” ratings are traditionally ignored in calculations of conditioned pain modulation). After one “foot and wrist” stimulation, the thermode attached to their wrist was removed, but the thermode attached to their foot remained attached for use in Phase 2. Thermal pain during “No VR” was used during Phase 1. VR was not used in Phase 1. Conditioned Pain Modulation (CPM) (involving two simultaneous stimuli) was only measured in Phase 1. No CPM during Phase 2. All stimuli during Phase 2 were single thermode.

### Phase 2: measuring whether physically touching real water makes virtual reality more effective at reducing pain: single thermode (foot) pain during two different VR conditions

During Phase 2, each participant received two separate single thermode stimuli (one at a time) to their “foot only” at the same baseline temperature used in Phase 1 (“VR with no real water” during one thermal stimulus, and “VR + real water” during the other single foot stimulus, with treatment order randomized using random number sequences from www.random.org). Blocked randomization with a block size of 2 was used. To reduce bias, the researcher only learned the sequential treatment order of an individual participant when the intervention treatment order was assigned. The PI generated the allocation sequence, enrolled the participants, and assigned participants to interventions.

### Touching virtual water only (no real water/no tactile feedback)

Participants placed a VR Head Mounted Display (HMD) on their head. The HMD blocked their view of the real world. In the HMD, in the current study, participants could look around and see themselves (virtual hands only), in a castle room in virtual reality and could see a large “visual only” computer generated virtual wooden bowl filled with animated virtual water within reach of their virtual hand. Wiggling their real fingers and/or moving their real hands correspondingly moved their virtual hands in VR, allowing participants to interact with virtual object images via their virtual hands. In this treatment condition of the current study, during brief thermal pain stimuli to the dorsal foot, while in VR, healthy volunteer participants put their virtual hand into the “visual only” bowl of computer generated animated water in the virtual world (their real hand simultaneously reached into the air in the real world during this “visual only” VR condition, “no real water”).

After receiving a brief thermal stimulus while in ”VR + No real water”, participants took off the VR HMD and answered the pain ratings, using the pen and paper GRS pain ratings, followed by sense of presence and embodiment ratings. The pain ratings and subsequent instructions phase lasted approximately 5 min, and served as the “wash out” period between VR treatments, to help minimize carryover effects.

### Mixed reality analgesia “visual + tactile” treatment (avatar VR + yes real water)

**Figure 3:**
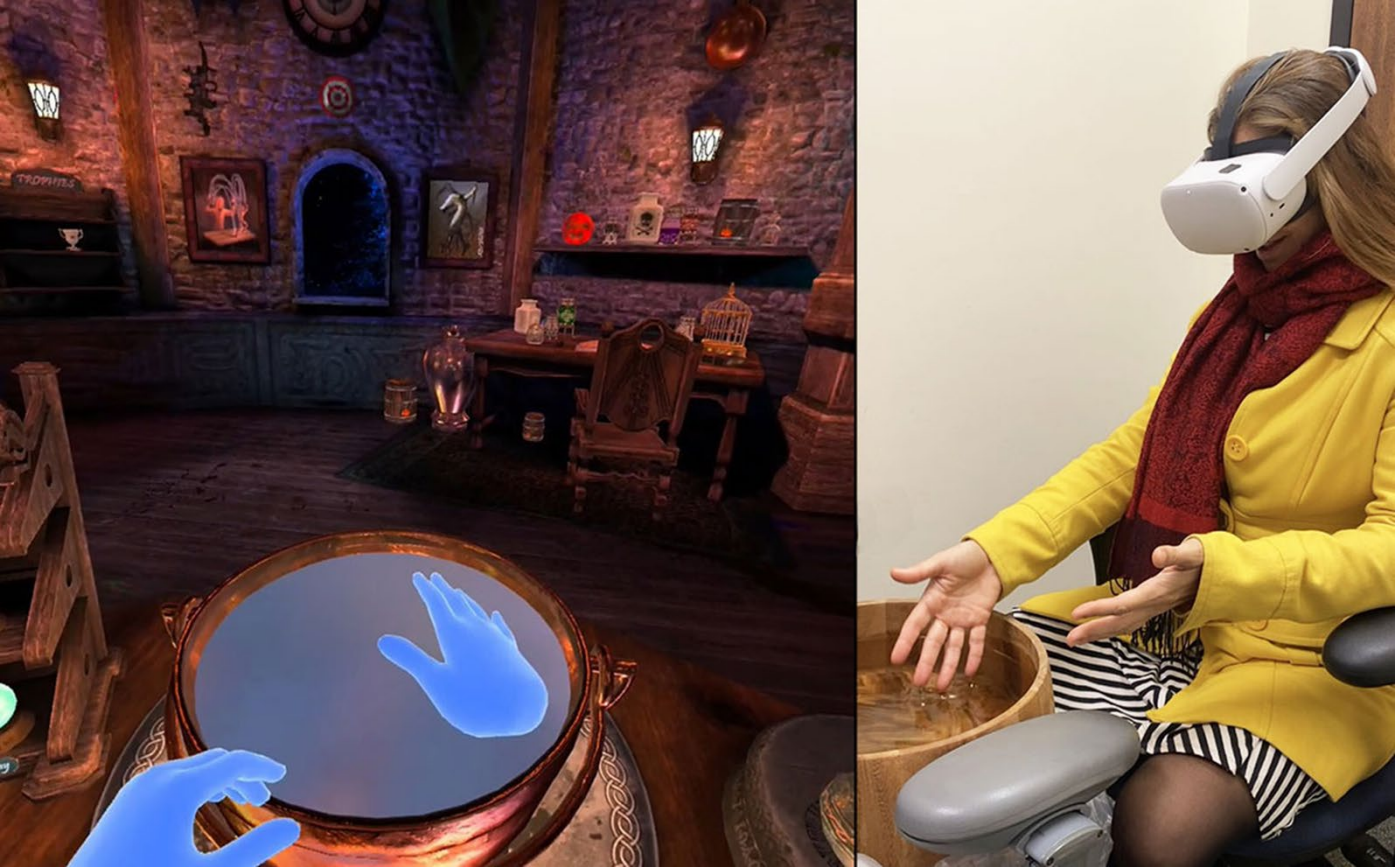
Mixed Reality. VR + real water. While wearing the Meta Quest2 VR HMD, using camera based hand tracking, any movements of the participant’s real hands or fingers in the real world are seen by the healthy volunteer participants in the virtual world. During the mixed reality condition, when their virtual hand went into a bowl of animated water in VR (left image), their real hand simultaneously went into a carefully positioned bowl of real water designed to give the computer generated virtual water tactile qualities (image on the right, right image copyright Hunter Hoffman, UW, https://www.vrpain.com). The image on the left is from the Waltz of the Wizards by Aldin Dynamics, Reykjavik, Iceland, https://www.aldin.io/, still shot copyright Hunter Hoffman, UW, https://www.vrpain.com).

During the interactive embodied virtual hand avatar VR analgesia condition with tactile feedback (VR + Yes real water, see Fig. 3) during a brief pain stimulus, participants touched the computer generated virtual water with their virtual hand, while their real hand simultaneously touched co-located wet liquid real water to give tactile feedback. Participants then took off the VR HMD and answered the pain ratings, using the pen and paper GRS pain ratings, followed by sense of presence and embodiment ratings.

For pain stimuli during virtual reality, in addition to answering questions 1–5 mentioned earlier (e.g., worst pain ratings), participants were also asked the following questions after each VR experience (see full questionnaire in "Appendix 1"). (6) To what extent (if at all) did you feel NAUSEA (sick to your stomach) as a result of experiencing the virtual world during the most recent VR session? (7) While experiencing the virtual world, to what extent did you feel like you WENT INSIDE the computer-generated world during the most recent VR session? The questions were answered on scales with the endpoints “not at all” to “very much” and were adapted from^28–32^. Similar “sense of presence” measures have been shown to be reliable and able to detect treatment effects^21,22,33^.

Further questions focused on embodiment and sense of agency and the participants perceived reality of the VR experience: While you were in VR, to what extent did you suspend disbelief and accept the idea that the virtual hands were your hands while you were in VR, “the virtual hands were my hands”. This scale was adapted from^34,35^. How much control did you have over the virtual hands? And how REAL did the virtual water seem to you during the most recent VR session? All questions were answered with GRS scales with the endpoints “not at all” to “very much”. After each VR + pain session (in Phase 2), participants answered all GRS questions about their pain and VR and avatar experiences.

### Phase 3: assessment of attention during VR conditions: does VR drain attentional resources?

Phase 3 (attention during VR) measured performance on a brief cognitive test designed to quantify how much attention was paid to the odd number task^22^ and how much attention was diverted by VR, by measuring the participant’s accuracy on the attention demanding auditory “odd number” task (1) during No VR, (2) during VR + no real water, and (3) during VR + yes real water (See Fig. 2). The VR treatment order was randomized. The traditional odd number divided attention task^36–39^ was adapted for use in the current VR study, using a new number set (see "Appendix 2") customized for the present study (with 10 odd number triads during a 2 min session)^22^. The researcher played the pre-recorded auditory string of numbers (see below) via a digital audio file on a laptop with high quality Dolby Sound laptop speakers. The string of numbers (i.e., the odd number task) lasted 2 min per session. The same identical 2 min audio file was played a total of three times. The first time, it was played with No VR (baseline). Participants did not wear a VR HMD during the No VR condition. They monitored the auditory odd number task with No VR. The second time the odd number task was played during “VR + No real water” (e.g., visual only computer generated virtual water). During this VR condition, participants went into a virtual room in an ancient castle, and were instructed to reach out and put their virtual hand into the visual image of a virtual bowl of animated water they saw in front of them in VR. While holding their virtual hand in the (visual only) computer generated virtual water, they also listened to an auditory string of numbers, and said “now” any time they heard three odd numbers in a row. And the third time, the odd number audio was played in VR again during VR + Yes real water (e.g., virtual water with tactile feedback). During this VR condition, participants went into a virtual room in an ancient castle, and were instructed to reach out and put their virtual hand into the bowl of animated water they saw in front of them in VR. In addition, a bowl of real water was placed in the appropriate location so when the participant’s virtual hand went into the computer generated bowl of virtual water in VR, their real hand went into the co-located bowl of real water in the real world (see Fig. 3). While holding their virtual hand in the virtual (and real) water, they also listened to an auditory string of numbers, and said “now” any time they heard three odd numbers in a row.

The VR treatment order used for the Phase 3 divided attention task was the opposite of the VR treatment order used in Phase 2 (thermal pain stimuli). Since the treatment order of Phase 2 was randomized, VR treatment order was also randomized in Phase 3. It is important to note that there were no thermal pain stimuli during the three odd number tasks.

During Phase 3, in addition to measuring participants accuracy on the odd number task, two new original GRS questions were introduced for the first time in the current study, to measure the participants subjective experience about how much VR interfered with their ability to do the odd number task. After completing all three of the two minute odd number sessions, participants were asked to rate/compare how distracting they found “No VR” versus how distracting they found “VR + No real water” versus how distracting they found “VR + Yes real water”. And they were then asked to rate how hard it was to concentrate on the odd numbers during “No VR” versus during “VR + No real water” versus during “VR + Yes real water” (see "Appendix 3" for details). They verbally told their answers to the researcher, who marked the GRS scales for the participants (using three different colored pens and labeling each response to keep the subject’s answers in each condition clear).

### Primary hypothesis (worst pain) power analysis

A power analysis to determine the number of participants needed to test our primary hypothesis was computed a priori, using the statistical program GPower 3.10^40^. The following assumptions were used in the power analyses, all determined from pilot data^19^: an effect size (d) of 0.78, power of 0.95, and an alpha of 0.05. Under these conditions, we would require only 24 participants to be able to detect a significant treatment effect, and to show that interactive virtual hands + tactile feedback (VR + Yes real water) was more effective than VR with no real water for reducing acute worst pain during the brief thermal pain stimuli. The power analysis calculates an estimation of the minimum required sample size for an experiment to achieve a specific statistical power and effect size (for the primary hypothesis), and collecting a pre-specified sample larger than minimum is ideal. The current (n = 48 subject) study used more subjects than the minimum sample identified by the power analysis (n = 24), in case the data showed more variance than estimated, and to have enough power to conduct secondary analyses (e.g., correlations, which often require extra power).

## Results

Since we had a small sample size, determining the distribution of the primary outcome variable (worst pain ratings under 3 treatment conditions, during No VR, during VR with no tactile feedback, and during VR with tactile feedback) was important for choosing an appropriate statistical method. Normality is an important assumption of parametric statics. Kolmogorov–Smirnov tests were performed and showed that the distribution of worst pain ratings departed significantly from normality for worst pain during No VR, D(48) = 0.157, *p* = 0.005, and departed significantly from normality for worst pain during no tactile feedback, D(48) = 0.148, *p* < 0.05, and departed significantly from normality for worst pain during VR with tactile feedback, D(48) = 0.182, *p* < 0.001. Based on this outcome, non-parametric statistics were used for all analyses.

Pre-analysis for carry-over effects. When using a within-subjects design, carry over effects sometimes occur when the effect of the first treatment condition persists, and influences the responses of the second treatment, so that the size of the observed difference between the treatment conditions depends on the treatment order^24^. As shown below, repeated measures Friedman comparisons were used to test if there were undesired carryover treatment order effects in the current study. The difference between visual only Avatar VR (VR + no real water) versus visual + tactile VR (VR + real water) was the dependent variable, and treatment order was the between-group factor. People who received visual only VR first were considered one group (n = 24), and those who received “visual only” second were considered a second group (n = 24), for the purpose of these pre-analyses of possible carry over effects. All participants were in the originally assigned treatment order. No significant carry over effects (interactions with treatment order) were found for Time spent thinking about pain, Z =  − 0.61, *p* = 0.54 NS; pain unpleasantness, Z =  − 1.04, *p* = 0.30 NS; Worst pain, Z =  − 0.94, *p* = 0.35 NS, Fun, Z =  − 0.66, *p* = 0.51 NS, or anxiousness =  − 1.79, z = 0.07 NS. Thus all of the following analyses were collapsed across treatment order (n = 48).

### Worst pain (primary outcome measure)

**Figure 4:**
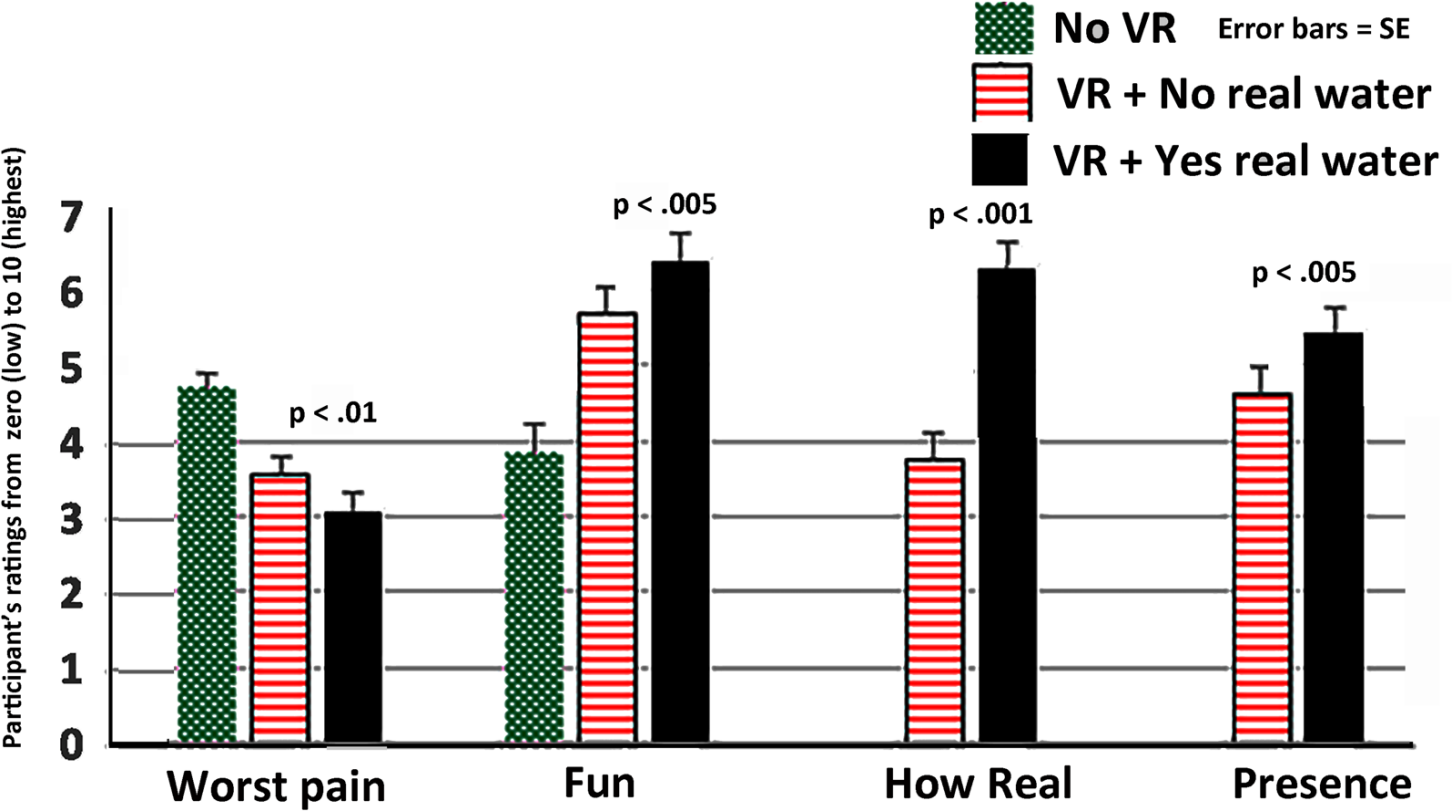
Physically touching virtual water significantly reduced participant’s worst pain ratings, and significantly increased fun, significantly increased how real the water seemed, and significantly increased the participant’s illusion of “being there” in VR (i.e., their sense of presence).

A Friedman test showed a significant main effect for Worst pain, χ2 (2) = 36.77, *p* < 0.001. Post hoc paired comparisons (Wilcoxon signed rank tests) for the variable “worst pain” are as follows (and see Table 2 and Fig. 4). (1) Comparing “No VR”, Mean = 4.71 (SD = 1.25) versus “VR + No real water”, Mean = 3.56 (SD = 1.63), worst pain was significantly lower during “VR + No real water”, Z = 4.33, *p* < 0.001, r = 0.6, a large effect size. (2) Comparing “No VR", mean = 4.71 (SD = 1.25) versus “VR + Yes real water”, Mean = 3.08 (SD = 1.65), worst pain was significantly lower during “VR + Yes real water”, Z = 4.86, *p* < 0.001, r = 0.7, a large effect size. And (3) Most importantly, as predicted, comparing “VR + No real water”, Mean = 3.56 (1.63) versus “VR + Yes real water”, Mean = 3.08 (SD = 1.65), worst pain was significantly lower during “VR + Yes real water”, Z = 2.66, *p* < 0.01, r = 0.4, a medium effect size.

**Table 2:** Worst pain, mean ratings on a zero to 10 graphic rating scale (SD in parentheses), where 10 = excruciating pain.

Worst pain	No VR (baseline)	VR no real water	VR yes real water	Wilcoxon signed rank tests	Effect size
“No VR versus VR + No real water”	Mean = 4.71 (SD = 1.25)	Mean = 3.56 (SD = 1.63)		Z = 4.43, *p* < 0.001	r = 0.6 large effect size
“No VR versus VR + Yes real water”	Mean = 4.71 (SD = 1.25)		Mean = 3.08 (SD = 1.65)	Z = 4.86, *p* < 0.001	r = 0.7, large effect size
“VR + No real water” versus “VR + Yes real water”		Mean = 3.56 (SD = 1.63)	Mean = 3.08 (SD = 1.65)	Z = 2.66, *p* < 0.01	r = 0.4, medium effect size

### Unpleasantness (secondary outcome measure)

A Friedman test showed a significant main effect of “No VR” versus “VR + No real water” versus “VR + Yes real water” for pain unpleasantness, χ2 (2) = 36.73, *p* < 0.001. Post hoc paired comparisons (Wilcoxon signed rank tests) for the variable “pain unpleasantness” are as follows. (1) Comparing “No VR”, Mean = 4.45 (SD = 1.29) versus “VR + No real water”, Mean = 3.08 (SD = 1.60), pain unpleasantness was significantly lower during “VR + No real water”, Z = 4.44, *p* < 0.001, r = 0.64, large effect size. (2) Comparing “No VR”, Mean = 4.45 (SD = 1.29) versus “VR + Yes real water”, Mean = 2.59 (SD = 1.57), pain unpleasantness was significantly lower during “VR + Yes real water”, Z = 5.40, *p* < 0.001, r = 0.78, large effect size. And (3), comparing “VR + No real water”, Mean = 3.08 (SD = 1.60) versus “VR + Yes real water”, Mean = 2.59 (SD = 1.57), pain unpleasantness was slightly but non-significantly lower during “VR + Yes real water”, Z = 1.94, *p* = 0.05 NS, (in the predicted direction), r = 0.28 small effect size.

### Time spent thinking about pain (a secondary pain measure)

A Friedman Test showed a significant main effect of “No VR” versus “VR + No real water” versus “VR + Yes real water” for Time spent thinking about pain, χ2 (2) = 31.52, *p* < 0.001. Post hoc paired comparisons (Wilcoxon signed rank tests) for the variable “Time spent thinking about pain” are as follows. (1) Comparing “No VR”, Mean = 4.52 (SD = 1.54) versus “VR + No real water”, Mean = 3.01 (SD = 1.78), Time spent thinking about pain was significantly lower during “VR + No real water”, Z = 3.98, *p* < 0.001, r = 0.57, large effect size. (2) Comparing “No VR”, Mean = 4.52 (SD = 1.54) versus “VR + Yes real water”, Mean = 2.59 (SD = 1.79), Time spent thinking about pain was significantly lower during “VR + Yes real water”, Z = 4.51, *p* < 0.001, r = 0.65, large effect size. Comparing “VR + No real water”, Mean = 3.01 (SD = 1.78) versus “VR + Yes real water”, Mean = 2.59 (SD = 1.79), “Time spent thinking about pain” was slightly but non-significantly lower during “VR + Yes real water”, Z = 1.34, *p* = 0.18 NS, (i.e., in the predicted direction), r = 0.19 small effect size.

### Fun

A Friedman Test showed a significant main effect of “No VR” versus “VR + No real water” versus “VR + Yes real water” for Fun during the thermal stimulus, χ2 (2) = 30.61, *p* < 0.001. Post hoc paired comparisons (Wilcoxon signed rank tests) for the variable “Fun” are as follows. (1) comparing “No VR”, Mean = 3.84 (SD = 2.54) versus “VR + No real water”, Mean = 5.69 (SD = 2.41), participants reported having significantly more fun during “VR + No real water”, Z = 4.40, *p* < 0.001, r = 0.63, large effect size. (2) Compared to “No VR”, Mean = 3.84 (SD = 2.54), “VR + Yes real water”, Mean = 6.39 (SD = 2.41) was significantly more fun, Z = 5.01, *p* < 0.001, r = 0.72, large effect size. And (3) Most importantly, compared to “VR + No real water”, Mean = 5.69 (SD = 2.41), “VR + Yes real water” Mean = 6.39 (SD = 2.41) was significantly more fun, Z = 3.23, *p* < 0.005, r = 0.47, a moderate effect size (see Fig. 4).

### Anxiousness

A Friedman Test showed a significant main effect of “No VR” versus “VR + No real water” versus “VR + Yes real water” for how anxious they felt during the thermal stimulus, χ2 (2) = 33.18, *p* < 0.001. Post hoc paired comparisons (Wilcoxon signed rank tests) for the variable “anxious” are shown as follows: (1) comparing “No VR”, Mean = 2.84 (SD = 2.04) versus “VR + No real water”, Mean = 1.56 (SD = 1.68), participants reported being significantly less anxious during “VR + No real water”, Z = 4.34, *p* < 0.001, r = 0.63, large effect size. (2) Compared to “No VR”, Mean = 2.84 (SD = 2.04) participants were significantly less anxious during “VR + Yes real water”, Mean = 1.44 (SD = 1.79), Z = 4.47, *p* < 0.001, r = 0.65, large effect size. Comparing “VR + No real water” versus “VR + Yes real water”, anxiety was only slightly lower during “VR + Yes real water”, Z = 0.43, *p* = 0.67 NS (non-significant but in the predicted direction), r = 0.06, small effect size.

### Sense of presence

Participants’ sense of presence ratings are as follows. Compared to their illusion of presence (being there) during “VR + No real water”, Mean = 4.64 (SD = 2.24), participants reported having a significantly stronger illusion of presence in virtual reality (“being there” in the virtual world), during “VR + Yes real water”, Mean = 5.44 (SD = 2.33) (where higher presence ratings are better, on a zero to ten rating scale), Z = 3.03, *p* < 0.005, r = 0.44 a moderate effect size (see Fig. 4). As predicted, we also found a statistically significant correlation between “sense of presence during VR + Yes real water” and “VR analgesia during VR + Yes real water”, (Pearson r = 0.30, *p* < 0.05).

### Embodiment

Compared to their ownership/embodiment of their avatar virtual hands, (i.e., the virtual hands were my hands) during “VR + No real water”, Mean = 5.19, (SD = 2.83) participants reported feeling a significantly stronger illusion of ownership/embodiment of their avatar virtual hands during “VR + Yes real water”, Mean = 5.94 (SD = 2.87), (where higher ratings indicate stronger embodiment), Z = 3.72, *p* < 0.001.

### Sense of agency

Compared to VR + No real water, Mean = 7.16 (SD = 2.20), participants reported no significant increase in control over their virtual hands during VR + Yes real water, (Mean = 7.36, SD = 2.17), Z = 1.61, *p* > 0.05 NS.

### How real was the virtual water?

Compared to the realness of the virtual water during visual only “VR + No real water”, Mean = 3.76 (SD = 2.34), the virtual water seemed significantly more real during “VR + Yes real water”, Mean = 6.30 (SD = 2.44) (where higher ratings indicate higher realism), Z = 5.28, *p* < 0.001, see Fig. 4.

### Nausea

Nausea during VR was near zero for both treatment conditions. Compared to nausea during VR + No real water, Mean = 0.39 (SD = 1.10), participants reported no significant increase in nausea during VR + Yes real water, (Mean = 0.42, SD = 1.31), Z = 0.07, *p* = 0.95 NS.

## Phase 3 results

We predicted participants would be less accurate on the odd number task during VR than during “No VR”, and would be least accurate during VR + Yes real water. A Friedman Test showed a significant main effect of “No VR” versus “VR + No real water” versus “VR + Yes real water” for accuracy (mean number of hits out of 10 possible hits) on the odd number task, χ2 (2) = 17.47, *p* < 0.001. Accuracy results, (mean number of hits on the odd number task out of 10 possible hits during each 2 min odd number session, e.g., 9/10 hits = 9.00 = 90% accurate, with SD in parentheses) are as follows.

As shown in Table 3, participants were 93% accurate (mean = 9.34 correct out of 10 possible, SD = 1.01) on the odd number task during “No VR”, but made significantly more errors on the odd number task during VR + no real water, 84% accurate (mean = 8.35, SD = 1.54), Z = 4.43, *p* < 0.001, r = 0.64, large effect size.

**Table 3:** Accuracy on the Odd number “divided attention” task.

Accuracy on the odd number divided attention test	No VR	VR + no real water	VR + yes real water	Wilcoxon signed rank test
“No VR versus VR + No real water”	9.34 (1.01) (93% accurate)	8.35 (1.54) (84% accurate)		Z = 4.43, *p* < 0.001
“No VR versus VR + Yes real water”	9.34 (1.01) (93% accurate)		8.19 (1.77) 82% accurate	Z = 3.91, *p* < 0.001
“VR + No real water” versus “VR + Yes real water”		8.35 (1.54) (84% accurate)	8.19 (1.77) (82% accurate)	Z = 0.89, p > 0.05 NS

We predicted participants would be less accurate during VR than during No VR, and would be least accurate during VR + Yes real water. Means (SD).

Similarly, participants were 93% accurate during No VR (mean = 9.34 correct, SD = 1.01), and participants made significantly more errors during VR + yes real water, 82% accurate (mean = 8.19, SD = 1.77), Z = 3.91, *p* < 0.001, r = 0.56, large effect size.

There was no significant difference in the accuracy on the odd number task during VR + No Real Water (M = 84% correct, mean = 8.35 SD = 1.54) versus VR + Yes real water 82% correct, mean = 8.19, (SD = 1.77), Z = 0.89, *p* > 0.05 NS, r = 0.14 small effect size.

Participants’ ratings of how distracting they found VR, and how difficult VR made it to concentrate, are as follows. Participants rated how distracting VR was during the Odd number divided attention task on a zero to 10 scale where 0 = “not distracting at all” and 10 = “extremely distracting” (higher numbers indicate more distraction).

We predicted participants would find VR subjectively more distracting than “No VR”, and especially during VR + Yes real water. The results were as follows: On a subjective rating scale from zero to ten (where higher numbers = more distracting), participants rated “VR + No real water” as Mean = 4.94 (SD = 2.02) moderately distracting, and significantly more distracting compared to “no distraction” during “No VR”, Mean = 0.75 (SD = 1.09), Z = 5.89, *p* < 0.001, r = 0.85, large effect size. Similarly, participants rated VR + Yes Real Water, Mean = 5.44 (SD = 1.71) moderately distracting, and significantly more distracting than “No VR”, Mean = 0.75, (SD = 1.09), Z = 5.86, *p* < 0.001, r = 0.85, large effect size. Finally, “VR + No real water”, Mean = 4.94 (SD = 2.02) was not significantly different from the participants subjective rating of how distracting they found “VR + Yes Real water”, Mean = 5.44 (1.71), Z = 1.39, *p* > 0.05, NS, r = 0.20 small effect size, but was in the predicted direction.

On a scale from zero to ten, participants rated their difficulty concentrating during “VR + No real water” as “moderately difficult”, Mean = 4.94 (SD = 1.79), which was significantly more difficult compared to “a little difficult” during “No VR”, (Mean = 1.14 (SD = 1.19), Z = 5.80, *p* < 0.001, r = 0.84, large effect size. Similarly, participants rated their difficulty concentrating during VR + Yes Real Water as “moderately difficult” to concentrate, Mean = 5.60 (SD = 1.99), and significantly more difficult to concentrate than “No VR” = 1.14 (1.19), Z = 5.77, *p* < 0.001, r = 0.83 large effect size. However, although in the predicted direction, “VR + No real water”, Mean = 4.94 (SD = 1.79) was not significantly different from the participants subjective rating of how difficult it was to concentrate during VR + Yes Real water = 5.60 (1.99), Z = 1.82, *p* = 0.07, NS, r = 0.26 small effect size.

### Conditioned pain modulation during Phase 1: (reported last for ease of exposition)

Phase 1 (note that VR was not used for any of the stimuli in Phase 1).

For worst pain ratings, as predicted, participants rated the worst pain they felt during “foot only” during a single stimulus of their foot as “moderate pain”, Mean = 4.74 (SD = 1.27), and the pain they felt on their foot dropped to “mild pain” Mean = 2.96 (SD = 1.91) when they simultaneously received stimulation of their foot and wrist at the same time, Wilcoxon Z = 4.64, *p* < 0.001 (i.e., significant Conditioned Pain Modulation, CPM), r = 0.67, large effect size.

Similarly, they rated ”pain unpleasantness” during “foot only” during a single stimulus of their foot as “moderately unpleasant” Mean = 4.49 (SD = 1.30), and this rating dropped to “mildly unpleasant” Mean = 2.76 (SD = 1.79) when they simultaneously received stimulation of their foot and wrist at the same time, Wilcoxon Z = 5.16, *p* < 0.001 (i.e., significant CPM), r = 0.75 large effect size.

Similarly, participants rated the amount of “time they spent thinking about their pain” during “foot only” during a single stimulus of their foot as approximately half of the time, Mean = 4.38 (SD = 1.50), and this rating dropped to “some of the time”, Mean = 2.70 (SD = 2.04) when they simultaneously received stimulation of their foot and wrist at the same time, Wilcoxon Z = 4.50, *p* < 0.001, (i.e., significant CPM), r = 0.65, large effect size.

### The correlation between VR analgesia and conditioned pain modulation

Analyses indicate a significant correlation between how much VR + Yes real water reduced worst pain ratings, and reduction in worst pain during CPM (Pearson r = 0.46, *p* < 0.005).

Similarly, in exploratory post-hoc analyses, participants with smaller than average CPM analgesia still showed a statistically significant (25% VR analgesia) reduction in worst pain during mixed reality: ”No VR” = 4.53, (SD = 1.31) versus VR + real water = 3.38, (SD = 1.59), Z = 2.79, *p* = 0.005, r = 0.40 moderate effect size.

The subset of participants with larger than average CPM during Phase 1 showed a much larger (46% VR analgesia) reduction in worst pain (i.e., greater VR analgesia) during mixed reality: (No VR Mean = 4.77, (SD = 1.13) versus VR + Yes real water (mean = 2.57, SD = 1.66), Z = 3.65, *p* < 0.001, 0.53 large effect size.

## Discussion

In the current study we used real water to give virtual objects (i.e., animated virtual water) more realistic physical properties (wet liquid qualities). As predicted, compared to reaching their virtual hand into the computer-generated virtual water with no tactile feedback (as their real hand reached into the air), adding real water to the immersive VR experience significantly increased VR analgesia (r = 0.5, a large effect size), increased the illusion of presence, increased avatar (virtual hand) embodiment, increased how much fun participants had during the pain stimulus, and increased how real the computer generated virtual water felt to the subjects. Compared to “No VR”, “VR + Yes Real Water” reduced worst pain by 35% in the current study. This 35% reduction in worst pain is consistent with our previous results showing that VR analgesia can be comparable to the opioid analgesia from a moderate dose of hydromorphone^18^. Similarly, a “No VR” study measuring opioid analgesia only^41^ also found approximately 33% reduction in experimental analog pain during a moderate dose of Dilaudid hydromorphone.

In the current study, as predicted, we also observed a significant correlation between how present participants felt during VR + real water, and how much their pain was reduced during VR + real water. In addition, on a divided attention task, going into VR during the odd number task significantly increased the number of errors made on the odd number task during each VR condition, and participants reported that they found it significantly more distracting and more difficult to concentrate on the odd number task during VR than performing the odd number task with “No VR”. Contrary to predictions, although in the predicted direction, adding tactile feedback did not significantly reduce accuracy on the odd number task, or participants’ ratings of how much VR distracted them during the odd number task.

In the current study, we measured conditioned pain modulation. Participants reported significant reductions in the worst pain they felt on their “foot only” (the target stimulus) when they also received a simultaneous thermal heat pain stimulus to their wrist, (a conditioning pain stimulus). This reflects a functioning endogenous pain modulation system. In addition to “pain reduces pain” reductions in worst pain (i.e., significant CPM for worst pain, a measure of the sensory component of pain), the current study found that CPM was also significant for pain unpleasantness (a measure of the emotional component of pain), and time spent thinking about pain (a measure of the cognitive component of pain). Interestingly, analyses indicate a significant correlation between how much VR + real water reduced worst pain ratings, and the reduction in worst pain during conditioned pain modulation.

According to Hoehn et al.^42^, CPM studies seldom control for the possibility that the second conditioning stimulus is simply distracting the patient’s attention away from their pain elicited by the target stimulus. Hoehn et al.^42^ recently found strong CPM results, controlling for distraction. Results of the current study showed there was a significant positive correlation between reduction of worst pain during CPM (during Phase 1), and VR analgesia (during mixed reality in Phase 2). Whether VR analgesia and CPM are related (i.e., share any common mechanisms), is an interesting question for future research. The reduction in accuracy on the attention demanding divided attention task (during VR in Phase 3 of the current study) implicates an attentional mechanism for how VR reduces pain in the current study^22^. In the literature, CPM is considered an important measure of the robustness of a person’s endogenous analgesia system^43^. For example, several studies have shown that CPM magnitude is lower in patients with chronic pain diseases, compared to the CPM of healthy controls^43^. The current results suggest that CPM may be due in part to distraction, and the correlation between CPM and VR analgesia raises questions about whether VR analgesia involves endogenous opioids (e.g., via endogenous inhibitory pathways), and whether chronic pain patients will show reduced VR analgesia for acute pain, compared to healthy controls. Encouragingly, in the present study, in exploratory analyses, participants with higher than average CPM scores showed much greater VR analgesia than participants with lower than average CPM scores, but participants with lower than average CPM scores still showed significant VR analgesia. If the current results generalize to chronic pain patients, we predict that chronic pain patients will show lower CPM and less VR analgesia than healthy college students, but chronic pain patients will still show large and significant reductions in pain during VR distraction, at least while they are in the VR HMD.

Overall, the results of this study implicate an attentional mechanism for how VR reduces pain. The results help understand how VR influences pain perception, and help guide the design of more immersive and more effective VR analgesia treatments in the future.

A number of experimental studies have now shown that increasing the immersiveness of the VR system increases the illusion of “being there” and/or increases VR analgesia for acute pain by increasing the field of view seen through the HMD, and by adding avatars and/or increasing interactions with visual only virtual objects^17,19,20,22,33^. In addition to measuring the participants sense of presence in the VR environment, the current study also measured how strong the sense of embodiment was in terms of virtual body (avatar) ownership (“these virtual hands are my hands”) and sense of agency (“how much control did you have over your virtual hands”). Following^22^ this study explored how the use of avatars increases the efficacy of VR in reducing acute pain. As noted by^15^ most previous immersive VR analgesia studies *involving avatars* have explored the use of VR for reducing chronic/persistent pain. In contrast, most VR analgesia studies using VR to reduce acute pain have not used avatars. For example, in SnowWorld^1,44^, during burn wound cleaning, children could look around and interact with the virtual world (e.g., via aiming virtual snowballs at virtual snowmen) but did not have virtual hands or other avatar representations (i.e., no virtual body while in VR). The current study is one of the first studies to explore the use of avatar VR to treat acute pain, see also^22^.

### Limitations

The current study has a number of limitations that should be taken into consideration when interpreting the results. The within-subjects design is statistically powerful and allows each participant to be compared for the different treatments, but has the drawback that in the current study, participants, care provider and primary outcomes assessor remain aware of the different treatment conditions, and this awareness has the potential to influence the results^45^. The current study should thus be replicated using a between-group design, ideally with participants blinded to VR treatment group conditions^46^ and using a much larger sample size.

Another limitation is that the current study is an analog experimental pain study, with only very brief thermal pain stimuli and brief VR treatment durations. Whether the current results generalize to clinical settings (e.g., for brief venipunctures^47,48^, or 5 min external fixator pin removal procedures for patients with a broken pelvic bone^14^, and ideally for 20 min burn wound care sessions^1,13,49^, is an important research topic for future clinical studies with patients during painful medical procedures. Previous non-pain studies have shown that interacting with one mixed reality object significantly increased participants perception of how real they found other virtual objects (e.g., touching a mixed reality ceramic dinner plate increased participants predictions about the solidity of the virtual walls^11,23^. Our previous early clinical mixed reality visual + tactile research with spider phobia patients showed that the mixed reality illusion can be very compelling and therapeutic^50,51^. Future research is needed on whether mixed reality can increase VR analgesia in clinical settings.

### Implications

In addition to implicating an attentional mechanism for how VR reduces pain, the results of this study could also have important clinical implications for how pain is treated in clinical practice^52^. According to^53^ the epidemic of opioid related overdose deaths in the USA and other countries^2,54^ has greatly increased the urgency to develop effective non-drug pain control techniques that can help reduce the medical community’s current heavy reliance on opioid analgesics for pain control. Although to date VR analgesia has typically been used adjunctively in addition to traditional pain medications, a stronger version of VR could reduce the use of opioids in some situations, e.g., patients who want to reduce/avoid potential risks of anesthesia side effects. e.g., having minor surgical procedures in the outpatient clinic with analgesia instead of the operating room with anesthesia^14^, and/or VR could be used to help compensate for increasing tendencies to under-medicate patients^55,56^, especially as opioids become much more strictly controlled, due to recent increases in federal regulation of opioid prescription^2^.

### Future directions

In addition to using VR for short term distraction from acute pain, there is growing interest in whether immersive virtual reality and/or augmented reality can be used to help reduce chronic pain, e.g., phantom limb pain or back pain^57,58^ see^15^ for a brief review). After having a limb amputated, most amputees experience painful sensations in the limb that is no longer there^59,60^. Both peripheral^61^ and central factors such as pathological cortical reorganization can contribute to phantom limb pain^62^. According to^63^, nearly one out of three amputees had the valuable subjective experience that their phantom limb would fuse with their plastic prosthesis when they were using it. The illusion that the plastic prosthesis is part of the patient’s body may help counteract phantom limb pain in amputees^64,65^. For example, in one survey of over 2000 limb amputees, patients reporting higher prosthesis ownership had significantly lower phantom limb pain^64^. We speculate that mixed reality illusions that facilitate prosthesis ownership (e.g., with the creative use of VR avatars) and/or that increase the illusion of embodiment during VR therapy sessions may help reduce phantom limb pain (e.g., in the 2/3rds of patients who perceive their prosthesis to be more of a tool than part of their own body, who have greater phantom limb pain than patients reporting prosthesis ownership). According to^65^, p 502, their results with phantom limb patients are consistent with animal studies suggesting that “behaviorally relevant tactile stimulation expands the cortical representation of the stimulated body region”.

There is some evidence that for some chronic pain patients (e.g., some phantom limb patients), VR therapy involving illusory embodiment of avatars may help reduce persistent pain, and may help normalize maladaptive pathological distortions of the patient’s homunculus in a subset of patients treated^57,66–69^. Mixed reality embodied avatars may be valuable for treating phantom limb pain and other chronic pain diseases, to create a more compelling illusion of limb ownership and to help treat phantom limb pain more effectively.

### Conclusions

The current results show that compared to No VR, physically touching virtual objects during immersive VR (tactile augmentation) reduced worst pain by 35%, consistent with our previous results showing that VR analgesia can be comparable to the opioid analgesia from a moderate dose of hydromorphone^18^. Tactile feedback also significantly increased avatar embodiment, the participants illusion of ownership of the virtual hands, which has potential to improve the effectiveness of avatar therapy for chronic pain in the future. Additional research and development is recommended.

## Supplementary Information

Supplementary Information 1.

Supplementary Information 2.

Supplementary Information 3.

## Data Availability

The datasets used and/or analyzed during the current study are available from the corresponding author on reasonable request.
