# Supplementary material for: Adding tactile feedback increases avatar ownership and makes virtual reality more effective at reducing pain in a randomized crossover study
Source: Sci Rep. 2023 May 22;13:7915. doi: 10.1038/s41598-023-31038-4 (PMC10203139; doi:10.1038/s41598-023-31038-4)
Supplement: Supplementary file 1 — Supplementary Information 1. [file 41598_2023_31038_MOESM1_ESM.docx]

Appendix 1, GRS Questionaire.

**A Magic Bowl Virtual Reality Analgesia for brief thermal pain**

**GRS**

Subject NUMBER___________CURRENT TIME: __________Date: _________

World: ___________

Current time and date of study: _________

Subjects Gender (Female or Male) Subjects age _______

Researcher’s name collecting this data today______________ _________

**Please indicate how you felt during the thermal heat stimulus you just received by CIRCLING or drawing a line through the appropriate number below. You can choose fractions, your answer does not have to be a whole number.**

1. How much TIME did you spend thinking about your pain during the most recent thermal stimulus you just received.

I THOUGHT about my pain……

|_______________________________________________|

0 1 2 3 4 5 6 7 8 9 10

None of some of half of most of All of

the time the time the time the time the time

2. How UNPLEASANT was your pain during the most thermal stimulus you just received.

|_______________________________________________|

0 1 2 3 4 5 6 7 8 9 10

not unpleasant mildly moderately severely excruciatingly

at all unpleasant unpleasant unpleasant unpleasant

3. Rate your WORST PAIN during the most recent thermal stimulus you just received.

|_______________________________________________|

0 1 2 3 4 5 6 7 8 9 10

no pain mild moderate severe excruciating

at all pain pain pain pain

4. How much FUN did you have during the most recent thermal stimulus you just received.

|_______________________________________________|

0 1 2 3 4 5 6 7 8 9 10

no fun mildly moderately pretty extremely

at all fun fun fun fun

5. Rate how anxious/nervous you were during the most recent thermal stimulus you just received.

|_______________________________________________|

0 1 2 3 4 5 6 7 8 9 10

Not anxious or nervous mildly moderately pretty extremely

at all anxious/nervous anxious/nervous anxious/nervous anxious/nervous

6. To what extent (if at all) did you feel NAUSEA (sick to your stomach) as a result of experiencing the virtual world during the most recent VR session?

|_______________________________________________|

0 1 2 3 4 5 6 7 8 9 10

no nausea mild moderate severe vomit

at all nausea nausea nausea

7. While experiencing the virtual world, to what extent did you feel like you WENT INSIDE the computer-generated world during the most recent VR session?

|_______________________________________________|

0 1 2 3 4 5 6 7 8 9 10

I did not feel mild moderate strong I went completely

like I sense of sense of sense of inside the

went inside going going going computer-

at all inside inside inside generated world

8. While you were in VR, to what extent did you suspend disbelief and accept the idea that the virtual hands were your hands while you were in VR.

|_______________________________________________|

0 1 2 3 4 5 6 7 8 9 10

I did not feel mild moderate strong I had

like they were sense of sense of sense of complete

my hands ownership ownership ownership ownership.

at all of the of the of the The virtual hands

virtual hands virtual hands virtual hands were my hands

while I was in VR while I was in VR while I was in VR while I was in VR while I was in VR

9. How much control did you have over the virtual hands?

|_______________________________________________|

0 1 2 3 4 5 6 7 8 9 10

I did not feel mild moderate strong I had

like I sense of sense of sense of complete

had any control control control control control

of the of the of the of the of the

virtual hands virtual hands virtual hands virtual hands virtual hands

10. How REAL did the virtual water seem to you during the most recent VR session?

|_______________________________________________|

0 1 2 3 4 5 6 7 8 9 10

completely somewhat moderately very Indistinguishable

fake real real real from real water
