# Supplementary material for: Adding tactile feedback increases avatar ownership and makes virtual reality more effective at reducing pain in a randomized crossover study
Source: Sci Rep. 2023 May 22;13:7915. doi: 10.1038/s41598-023-31038-4 (PMC10203139; doi:10.1038/s41598-023-31038-4)
Supplement: Supplementary file 2 — Supplementary Information 2. [file 41598_2023_31038_MOESM2_ESM.docx]

Appendix 2. After Phase 2, the thermal pain stimulator was removed from the top of the participant’s foot, and participants were told that Phase 2 was now over, and they received the following instructions. “You have just completed Phase 2 and we will now remove the thermal pain stimulator from your foot. Please take off the thermal stimulator and put your shoe back on now. There are no more pain stimuli, but we will now begin Phase 3 of the study. In Phase 3, no pain stimuli will be administered. Instead you will go into virtual reality again and while you are in the virtual world, you will monitor a string of numbers from 1 to 10, and will say ‘now’ any time you hear three odd numbers in a row, (the odd numbers are 1, 3, 5, 7 or 9). For example, if you hear “1, 9, 3”, you would say “now”. If you hear “1 9 4” you will not say now, because 4 is an even number. If you hear “1 9 4 3” you would still not say now, because you need 3 odd numbers in a row. Any time you hear an even number, your counter goes back to zero, and you need 3 odd numbers in a row. The researcher will keep track of your answers and will be measuring your accuracy on the odd number task. You will receive a total of three brief divided attention tasks. The first odd number session is with No VR. Are you ready to begin the odd number task? Just say “now” any time you hear 3 odd numbers in a row.”

1 2 3 5 7 5 2 4 1 9 3 6 5 8 1 1 4 3 5 1 2 3 2 1 7 9 2 5 2 2 1 9 5 6 2 4 6 7 1 8 2 6 4 7 5 3 1 6 7 2 1 9 3 5 1 8 2 1 5 3 8 1 3 2 9 7 6 5 3 1 3 5 1 4
