# Supplementary material for: Adding tactile feedback increases avatar ownership and makes virtual reality more effective at reducing pain in a randomized crossover study
Source: Sci Rep. 2023 May 22;13:7915. doi: 10.1038/s41598-023-31038-4 (PMC10203139; doi:10.1038/s41598-023-31038-4)
Supplement: Supplementary file 3 — Supplementary Information 3. [file 41598_2023_31038_MOESM3_ESM.docx]

Appendix 3

A Magic Bowl Virtual Reality Analgesia for brief thermal pain

GRS

Subject NUMBER___________CURRENT TIME: __________Date: _________

World: ___________

Current time and date of study: _________

Subjects Gender (Female or Male) Subjects age _______

Researcher’s name collecting this data today______________ _________

**Please indicate how you felt during the odd number task you just received by CIRCLING or drawing a line through the appropriate number below. You can choose fractions, your answer does not have to be a whole number.**

1. During the odd number task, how DISTRACTING was

a) No VR

b) VR with no real water

c) VR with real water

|_______________________________________________|

0 1 2 3 4 5 6 7 8 9 10

not distracting a little moderately very extremely

at all distracting distracting distracting distracting

2). During the odd number task, how hard was it to concentrate on the odd number task?

a) No VR

b) VR with no real water

c) VR with real water

|_______________________________________________|

0 1 2 3 4 5 6 7 8 9 10

not difficult a little moderately very extremely

at all difficult/hard difficult/hard difficult/hard difficult/hard

to concentrate to concentrate to concentrate to concentrate to concentrate
